# Circular RNA circCCDC9 acts as a miR-6792-3p sponge to suppress the progression of gastric cancer through regulating CAV1 expression

**DOI:** 10.1186/s12943-020-01203-8

**Published:** 2020-05-09

**Authors:** Zai Luo, Zeyin Rong, Jianming Zhang, Zhonglin Zhu, Zhilong Yu, Tengfei Li, Zhongmao Fu, Zhengjun Qiu, Chen Huang

**Affiliations:** 1 Department of General Surgery, Shanghai General Hospital, Shanghai Jiaotong University School of Medicine, 650 Xinsongjiang Road, Songjiang District, Shanghai, 201600 China; 2 Department of Gastrointestinal Surgery, Henan Provincial People’s Hospital, Zhengzhou, China

**Keywords:** CircCCDC9, miR-6792-3p, CAV1, Gastric Cancer

## Abstract

**Background:**

As a novel type of noncoding RNAs, covalently closed circular RNAs (circRNAs) are ubiquitously expressed in eukaryotes. Emerging studies have related dysregulation of circRNAs to tumorigenesis. However, the biogenesis, regulation, function and mechanism of circRNAs in gastric cancer (GC) remain largely unclear.

**Methods:**

The expression profile of circRNAs in 6 pairs of GC tissues and adjacent non-tumor tissues was analyzed by RNA-sequencing. Quantitative real-time PCR was used to determine the expression level of circCCDC9 in GC tissues and cell lines. Then, functional experiments in vitro and in vivo were employed to explore the effects of circCCDC9 on tumor growth and metastasis in GC. Mechanistically, dual luciferase reporter, fluorescence in situ hybridization (FISH), RNA immunoprecipitation (RIP) and RNA pull-down assays were performed to confirm that circCCDC9 directly sponged miR-6792-3p and alleviated suppression on target CAV1 expression.

**Results:**

Evidently down-regulated expression of circCCDC9 was observed in both GC tissues and cell lines. Expression of circCCDC9 was negatively correlated with tumor size, lymph node invasion, advanced clinical stage and overall survival in GC patients. Functionally, overexpression of circCCDC9 significantly inhibited the proliferation, migration and invasion of GC cell lines in vitro and tumor growth and metastasis in vivo, whereas miR-6792-3p mimics counteracted these effects. Mechanistic analysis demonstrated that circCCDC9 acted as a “ceRNA” of miR-6792-3p to relieve the repressive effect of miR-6792-3p on its target CAV1, then suppressed the tumorigenesis of GC.

**Conclusions:**

CircCCDC9 functions as a tumor suppressor in inhibiting the progression of GC through miR-6792-3p/CAV1 axis, which has provided an exploitable biomarker and therapeutic target for patients with GC.

## Introduction

Gastric cancer is known as the third most common malignancy worldwide with an estimated over 782,000 deaths in 2018 [1]. Owing to the lack of obvious symptoms and screening programs, most patients were found with advanced gastric cancer during the first diagnosis [2]. Although the therapeutic effects of GC have been improved so far, the prognosis of patients with GC remained largely unsatisfactory [3]. Therefore, further exploration of the molecular mechanisms underlying tumorigenesis and progression of GC is in urgent need to develop more efficient therapeutic strategies.

CircRNAs are a rediscovered class of endogenous non-coding RNAs within eukaryotes, which are typically comprised of exons and characterized by a covalently closed continuous loop and a canonical splicing junction site [4, 5]. Traditionally, they were regarded as meaningless by-products of processes in gene rearrangements and splicing [6]. Nevertheless, due to advances in high-throughput sequencing technologies and bioinformatics analysis, emerging studies have revealed circRNAs are generally stable, conserved and low-expressed and varies with different cell types and tissue types in expression level. Also, circRNAs are of multiple functions, including acting as miRNA sponges, binding to RNA-binding protein (RBP) and involving in protein translation [7–10]. In addition, circRNAs could participate in the initiation and development of multiple cancers and exert various influences on biological processes, including proliferation, invasion and metastasis [11, 12]. For example, circNHSL1 served as an oncogenic circRNA in progression and metastasis of gastric cancer through the circNHSL1/miR-1306-3p/SIX1/Vimentin regulatory gene network [11]; circDONSON promoted progression and development of GC cells via the NURF complex-dependent activation of SOX4 signaling [12]. However, the roles and functions of circRNAs in GC have not been fully elucidated.

MicroRNAs (miRNAs) are a class of pervasive, conserved small non-coding RNAs that act as negative gene regulators to repress the expression of target genes [13]. They involve a wide range of biological processes in cancers, such as proliferation, invasion, differentiation and apoptosis [14, 15]. Recent studies have reported that circRNAs could act as miRNA sponges by competitively binding with miRNA response elements (MREs) to inhibit their expression and function [16, 17]. For example, circMTO1 acts as a sponge of miR-9 to suppress the progression of hepatocellular cancer [18]. However, little is known about the circRNA-miRNA regulatory network in the progression of GC.

Caveolin-1 (CAV1) acts as a major scaffold protein of caveolae, which is omega-shaped vesicular invaginations of cell membrane [19]. Various classes of signaling molecules localized in the caveolae are negatively regulated by CAV1 through its “caveolin-scaffolding domain” (CSD) [20]. Previous studies demonstrated that CAV1 could recruit β-catenin to caveolae membranes. Then, recruited β-catenin could combine with E-cadherin to form cadherin-catenin complexes, which could promote cell adhesion and inhibit tumor metastasis [21–23]. Meanwhile, CAV1 could block the EGF-induced β-catenin-mediated transactivation and repress the transcription of cyclinD1, thus suppressing the cell cycle progression and tumor proliferation [24–26]. Moreover, results from our early studies revealed that CAV1 acted as a critical role in the progression and metastasis of pancreatic cancer [27, 28] and gastric cancer [29, 30]. However, the upstream regulation mechanism of CAV1 is still vague.

Here, based on the results of RNA-seq, we first identified a novel GC-related circRNA termed circCCDC9 derived from exons 6 and 7 of the CCDC9 gene with a circBase ID of hsa_circ_0000944. Subsequently, to obtain insights into the function and underlying molecular mechanism of circCCDC9 in the development and progression of GC, the clinical significance of circCCDC9 was explored in GC tissues. CircCCDC9 was down-regulated in GC tissues and acted as a sponge of miR-6792-3p to affect the expression of CAV1, and eventually modulated the tumorigenesis and progression of GC cells. Collectively, our findings have provided insights into the prognosis prediction and therapeutic target of GC.

## Methods

### Patient tissue samples and cell lines

There were two groups of gastric cancer and paired adjacent normal tissues in our study. One group contained fresh-frozen GC tissues and corresponding adjacent normal gastric epithelial tissues, which were obtained from 48 patients who underwent gastrectomy at Shanghai General Hospital between 2015 and 2018 in accordance with the Helsinki Declaration. After surgical resection, all specimens were frozen in liquid nitrogen and stably stored at − 80 °C until RNA extraction. The other group included 57 paired gastric cancer and matched normal tissues from 2013 to 2014, which were collected from patients who had underwent primary surgical treatment for GC in Shanghai General Hospital and immediately fixed with formalin. Then, these samples were embedded in paraffin to construct tissue microarray (TMA) and the final TMA contained 54 paired gastric cancer samples. All clinicopathological diagnoses were confirmed by two pathologists according to the 8th edition of the American Joint Commission on Cancer (AJCC) and the Union for International Cancer Control (UICC). The study was approved by the Ethics Committee of Shanghai General Hospital and informed consents were obtained from all patients.

The normal human gastric epithelial mucosa cell line (GES-1), and human GC cell lines (AGS, BGC-823, HGC-27, MGC-803, MKN-28, MKN-45, SGC-7901) were all purchased from the Culture Collection of Chinese Academy of Sciences (Shanghai, China). All cells were maintained and stored following the instructions obtained from their providers. Briefly, AGS, BGC-823, HGC-27, MGC-803, MKN-28, MKN-45 and SGC-7901 were maintained in RPMI-1640 medium (Gibco, USA) and HEK-293 T cell was maintained in DMEM medium (Gibco, USA), respectively. Both medium were supplemented with 10% fetal bovine serum (Gibco, USA), 1% penicillin and streptomycin (Gibco, USA). All cell lines were cultured in a humidified incubator containing 5% CO_2_ at 37 °C.

### RNA extraction and quantitative real-time polymerase reaction (qRT-PCR)

The total RNAs of the GC tissues/cell lines and corresponding normal tissues/cell lines were extracted using TRIzol Reagent (Takara, Japan) and genomic DNAs (gDNA) of those were isolated with FastPure DNA Isolation (Vazyme, China), according to the manufacturer’s instructions. The concentration and purity of RNA samples were measured by Nanodrop 2000 (Thermo Fisher Scientific, USA). For circRNA and mRNA, reverse transcriptions were performed using the PrimeScript RT Master Mix (Takara, Japan) with random primers. For miRNA, reverse transcriptions were performed using the PrimeScript RT Reagent Kit (Takara, Japan) with specific stem-loop primers. And cDNA amplification was performed using TB Green Premix Ex Taq II (Takara, Japan) with an ABI Prism 7500 sequence detection system (Applied Biosystems, USA). GAPDH and U6 were used as internal control, and each sample was repeated three times. Relative quantification of circRNA, miRNA and mRNA expression was compared to internal control and analyzed using the 2^-ΔΔCT^ method. Divergent primers were used to detect backsplice junction of circRNA and convergent primers were used to detect linear mRNA. The primers were listed in Additional file 1: Table S1.

### Nucleic acid electrophoresis

The cDNA and gDNA PCR products were investigated using 2% agarose gel electrophoresis with TAE running buffer. DNA was separated by electrophoresis at 100 V for 30 min. The DNA marker was Marker L (50-500 bp) (Sango Biotech, China). The bands were examined by UV irradiation.

### RNase R treatment

The RNAs (10 μg) from AGS and MKN-45 cells were treated with RNase R (3 U/μg, Epicenter) and incubated for 30 min at 37 °C. Then, the treated RNAs were reverse transcribed with divergent primer or convergent primer and detected by qRT-PCR assay and PCR assay followed by nucleic acid electrophoresis.

### Oligonucleotide transfection

MKN-45 and AGS cells were seeded in 6-well plates and cultured to 60–70% confluence before transfection. Si-circCCDC9, miR-6792-3p mimics, miR-6792-3p inhibitor and negative control oligonucleotides were designed and synthesized by GenePharma (Shanghai, China). Lipofectamine™2000 reagent (Invitrogen, USA) was used as transfection medium according to the manufacturer’s protocol. Oligonucleotide sequences were listed in Additional file 1: Table S2.

### Vector construction and stable transfection

To construct stable overexpression vector of circCCDC9, the full-length circCCDC9 cDNA was synthesized and cloned into overexpression vector pLCDH-ciR, which contained a front and back circular frame to promote RNA circularization. The empty vector with no circCCDC9 sequence was used as negative control. Stable transfection in AGS cells was performed as previously shown [31].

### Luciferase reporter assay

The sequences of circCCDC9 and CAV1–3’UTR and their corresponding mutation were designed, synthesized and inserted into luciferase reporter vector GP-miRGLO (Genepharma, China), termed circCCDC9-WT, circCCDC9-MUT, CAV1–3’UTR-WT and CAV1–3’UTR-MUT, respectively. All these plasmids were co-transfected with miR-6792-3p mimics or inhibitor to MKN-45 or AGS cell, respectively. Then, the relative luciferase activity was examined by Dual Luciferase Assay Kit (Promega, USA) in line with the manufacturer’s protocol.

### Biotin-coupled probe RNA pull down assay

Biotinylated circCCDC9 and miR-6792-3p (GenePharma, China) pull down assays were performed as described earlier [9]. In brief, 1 × 10^7^ GC cells were harvested, lysed, and sonicated. The probe was incubated with probes-M280 streptavidin dynabeads (Invitrogen, USA) at 25 °C for 2 h to generate probe-coated beads. The cell lysates were incubated with the probe-coated beads mixture at 4 °C overnight. After washing with the wash buffer, the RNA complexes bound to the beads were eluted and purified with Trizol Reagent (Takara, Japan) for further analysis. The sequences were listed in Additional file 1: Table S2.

### RNA immunoprecipitation

RNA immunoprecipitation (RIP) assay was performed using Magna RIP™ RNA-binding protein immunoprecipitation kit (Millipore, USA) following the manufacturer’s protocol. MKN-45 cells were lysed in complete RNA immunoprecipitation lysis buffer after transfected with miR-6792-3p mimics or negative control. Then, the cell extract was incubated with magnetic beads conjugated with anti-Argonaute 2 (AGO2) or anti-IgG antibody (Millipore, USA) for 6 h at 4 °C. The beads were washed and incubated with Proteinase K to remove proteins. Finally, isolated RNA was extracted using TRIzol Reagent (Takara, Japan), then, the purified RNA was subjected to agarose gel electrophoresis and qRT-PCR analysis.

### Fluorescence in situ hybridization (FISH)

FISH assay was executed to observe the location of circCCDC9 and miR-6792-3p in GC cells. Cy3-labeled circCCDC9 probes and FAM-labeled miR-6792-3p were designed and synthesized by GenePharma (Shanghai, China). Hybridization was performed overnight with circCCDC9 and miR-6792-3p probes according to the manufacturer’s instructions. The images were acquired on Zeiss LSM710 Laser Scanning Confocal Microscope (Zeiss Instrument Inc., Germany). Similarly, FISH assay was also performed in TMA, which contained 54 paired gastric cancer samples by Cy3-labeled miR-6792-3p (Boster, Shanghai). The intensity of staining was scored and termed as expression level by multiplying the intensity score (0 = negative, 1 = low, 2 = high) and the percentage of the total cell population (0 = none stained, 1 = 1 ~ 24% stained, 2 = 25 ~ 49% stained, 3 = 50 ~ 74% stained, 4 = 75 ~ 100% stained). The sequences of circCCDC9 and miR-6792-3p probe for FISH were listed in Additional file 1: Table S2.

### In situ hybridization (ISH)

The relative expression of circCCDC9 in GC tissues was detected by in situ hybridization with a specific digocin-labeled circCCDC9 probe (Digoxin-5′-CGCTCCTCCCACTCCTGCCGCTCGT-3′- Digoxin) (Geneseed, China) on TMAs containing 54 paired paraffin-embedded GC tissues. Briefly, after dewaxing and rehydration, the samples were digested with proteinase K, fixed in 4% paraformaldehyde, and hybridized with the digoxin-labeled circCCDC9 probe overnight, then incubated with anti-Digoxin mAb (Roche, Switzerland) at 4 °C overnight. The tissues were stained with NBT/BCIP and observed. The staining scores were scored and termed as expression level by multiplying the intensity score (0 = negative, 1 = low, 2 = high) and the percentage of the positively stained cells (0 = none stained, 1 = 1 ~ 24% stained, 2 = 25 ~ 49% stained, 3 = 50 ~ 74% stained, 4 = 75 ~ 100% stained).

### Cell counting kit-8 proliferation assay

A total of 3000 cells were plated in each well of a 96-well plate. Then, on the indicated time (24 h, 48 h, 72 h, 96 h), 10 μL of CCK-8 reagent (Dojindo Crop, Japan) was added directly into the culture medium. Then, the cells were incubated for 2 h at 37 °C, and the optional density (OD) was measured at 450 nm using a microplate reader (BioTek Instruments, USA). These experiments were repeated for three times.

### Colony formation assay

Transfected cells were seeded in 6-well plates at a density of 800 cells per well and then cultured at 37 °C in 5% CO_2_ for 2 weeks. Then, the cells were washed with phosphate-buffered saline (PBS), fixed with 4% paraformaldehyde for 20 min and stained with a 0.5% crystal violet solution for another 20 min. The colonies were then counted and analyzed. These experiments were repeated for three times.

### 5-Ethynyl-20-deoxyuridine (EdU) incorporation assay

The EdU assay was performed to assess the proliferation of cells by using a Cell-Light EdU DNA Cell Proliferation Kit (RiboBio, Guangzhou, China) following the manufacturer’s protocol. GC cells were cultured for 24 h in 24-well plates. These two cell lines were fixed using 4% paraformaldehyde after incubation with 50 mM EdU solution for 2 h. Then, cell lines were sealed with Apollo Dye Solution and Hoechst 33342 in sequence. The EdU cell lines were photographed and counted under an Olympus FSX100 microscope (Olympus, Tokyo, Japan). These experiments were repeated for three times.

### Wound healing assays

GC cells were cultured in six-well plates, and cell monolayer was subsequently scratched with a 200ul pipette tip. Representative images of cell migration were captured by photographing 10 high-power fields at 0 h and 24 h after injury. Remodeling was measured as the diminishing distance across the induced injury area normalized to the 0 h control and expressed as a relative migration rate. For each one, the experiments were repeated for at least three times with three replicates.

### Transwell migration and invasion assays

For Transwell assays, according to the manufacturer’s protocol, GC cells were seeded in upper chambers with 200ul of serum-free medium. The transwell chamber (Corning, USA) was paved with matrigel mix (BD Biosciences, USA) for invasion assays and paved without matrigel mix for migration assays. The bottom chamber was filled with medium and 10% FBS as a gastric cancer cell chemoattractant. After incubation for 24 h, the upper chambers were fixed and then stained by crystal violet (Kaigen, China) for 15 min. For visualization, the cell lines were photographed and counted in different five fields. These experiments were repeated for three times.

### Western blot

Cells were lysed in Radio Immunoprecipitation Assay lysis buffer (RIPA, Beyotime, China). The protein was prepared and quantified by bicinchoninic acid (BCA) analysis (Beyotime, China). The same amounts of protein were extracted by 10% SDS-PAGE and transferred onto polyvinylidene fluoride (PVDF) membranes (Millipore, MA, USA). The blocked protein with 5% skim milk powder was incubated with primary antibody anti-CAV1 (1:10000, abcam, MA, USA), anti-β-catenin (1:1000, Cell Signaling Technology, MA, USA), anti-cyclin D1 (1:1000, Cell Signaling Technology, MA, USA), anti-E-cadherin (1:1000, Cell Signaling Technology, MA, USA) and anti-GAPDH (1:5000, Cell Signaling Technology, MA, USA) at 4 °C overnight. Then the prepared membranes were incubated with secondary antibody (1:5000, Cell Signaling Technology, MA, USA) for 2 h. Finally, the blots were visualized by ECL chemiluminescent reagent (Millipore, Germany) and related data was analysed by Image Lab Softwave.

### IHC examination

Tissue samples were fixed in 4% paraformaldehyde embedded in paraffin, and sectioned. The tissue sections were incubated with anti-CAV1, anti-β-catenin, anti-E-cadherin, anti-cyclin D1 and anti-Ki-67 primary antibodies at 4 °C overnight and then incubated with an HRP-conjugated secondary antibody.

### Xenografts in mice

All animal care and experiments were performed according to the guidelines of the National Institutes of Health and approved by the Animal Care Committee of Shanghai General Hospital. We chose 4-week-old male BALB/c nude mice for tumor xenografts experiments to study the effect of circCCDC9 on tumor growth and metastasis. In tumor growth assay in vivo, AGS cells stably transfected with circCCDC9-overexpressing or control vector were subcutaneously injected into the upper back of the nude mice (1 × 10^7^, 150 μl). Volumes of tumors were measured weekly with a caliper. The tumor volume was calculated as (length×width^2^)/2. Finally, mice were sacrificed and subcutaneous tumor tissues were detected for tumor weight, WB and IHC staining. In tumor metastasis in vivo, circCCDC9-overexpressing or control AGS cells (5 × 10^6^) were intravenously injected into ileocolic vein of nude mice [32]. After 30 days, the livers were removed, paraffin-embedded and finally validated by hematoxylin and eosin (H&E) staining.

### Statistical analysis

Statistical analyses were performed using SPSS 22.0 (IBM, SPSS, Chicago, USA) and GraphPad Prism 7. Student’s t-test and one-way ANOVA were performed to analyze whether two or more groups had statistical significance. The Pearson correlation coefficient was used to analyze the correlations. The results were presented as the mean ± SD. OS curves and DFS curves were calculated with the Kaplan-Meier method and analyzed with the log-rank test. For all analyses, group differences were considered statistically significant for *P* < 0.05 between groups.

## Results

### Expression profiles of circRNA in GC tissues and paired normal gastric tissues

**Fig. 1:**
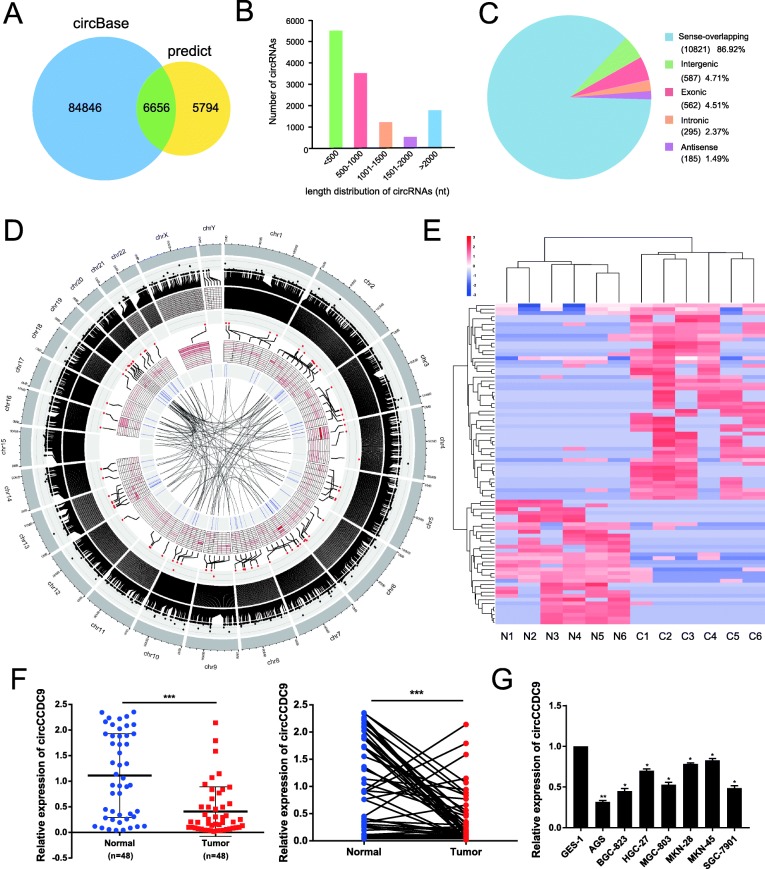
Expression profiles of circRNA in GC tissues and cell lines. **a**. Schematic illustration exhibiting overlap of circRNAs in RNA-seq (right) and circBase (left). **b**. Number of diverse length distributions of circRNAs. **c**. Composition of the detected circRNAs in terms of genomic origin. **d**. Circos plot displayed the distribution and expression of circRNAs on human chromosomes. The outermost layer was a chromosome map of the human genome. The inner circles from outside to inside corresponded to distribution and expression of detected circRNAs on the chromosomes, distribution and expression of significantly expressed circRNAs and predicted miRNAs sponged by significantly expressed circRNAs, respectively. **e.** A cluster heat map presented the significantly dysregulated circRNAs in GC tissues relative to matched adjacent normal tissues. The red and blue strips represented high and low expression, respectively. **f**. Relative expression of circCCDC9 in GC tissues and matched adjacent normal tissues was detected by qRT-PCR (*n* = 48). **g**. Relative expression of circCCDC9 in cell lines was determined by qRT-PCR. Data were showed as mean ± SD, *P < 0.05, **P < 0.01, ***P < 0.001

Numerous studies showed that circRNAs had assumed a pivotal role in the progression of cancers [11, 12, 16, 18]. To investigate the role of circRNAs in the progression of GC, we performed RNA-seq analyses of ribosomal RNA-depleted total RNA obtained from 6 clinical gastric cancer tissues and their paired adjacent normal tissues and constructed a circRNA profiling database. We detected 12,450 distinct circRNAs in all. Among them, 6656 circRNAs had been reported in circBase (Fig. 1a). The number of diverse length distribution of circRNAs was different, and the length of most circRNAs was less than 1000 nucleotides (Fig. 1b). Moreover, we analysed the composition of the significantly expressed circRNAs in terms of genomic origin (Fig. 1c). Circos plot displayed the distribution and expression of detected and significant expressed circRNAs on human chromosomes, as well as predicted miRNAs sponged by significantly expressed circRNAs (Fig. 1d). A cluster heat map presented the significantly dysregulated circRNAs in GC tissues compared to the matched adjacent normal tissues (Fold change> 5, *P* < 0.05) (Fig. 1e). Among the 85 differentially expressed circRNAs, 52 were upregulated and 33 were downregulated in GC tissues relative to normal tissues. Diluted by proliferation, circRNAs were often downregulated in cancers in which cell proliferation rates were high [33]. So we focused on the most downregulated circRNAs and matched them with circBase. Hsa_circ_0000944 (termed circCCDC9 in the remainder of the article), which was the most downregulated circRNA in our RNA-seq data, had attracted our attention. To further verify whether the expression level of circCCDC9 was downregulated in GC tissues according to the RNA-seq data, we detected downregulation of circCCDC9 in 48 GC tissues relative to adjacent normal tissues via qRT-PCR (Fig. 1f), which was consistent with the RNA-seq data. Moreover, circCCDC9 expression was then detected in gastric cancer tissues by ISH using TMA of 54 pairs of gastric cancer and adjacent normal tissues. Results of ISH indicated that circCCDC9 was significantly downregulated in gastric cancer compared to paired adjacent normal tissues (Fig. S1A and B). Further analysis showed that circCCDC9 was markedly lower in gastric cancer tissues of N1, N2, N3 (S1C), GTD ≥5 cm (S1D) and stage III (S1E) than those with N0, GTD < 5 cm and stage II, respectively (Fig. S1C-E). There was a negative correction between circCCDC9 expression and tumor size, lymph node invasion and TNM stage (Table S3). Kaplan-Meier survival curve also showed an positive correlation between the expression level of circCCDC9 and the prognosis of GC patients; exactly, GC patients with high circCCDC9 expression had longer OS and DFS (Fig. S1F and G).

Next, the expression levels of circCCDC9 were confirmed in the AGS, BGC-823, HGC-27, MGC-803, MKN-28, MKN-45 and SGC-7901 cell lines relative to GES-1 cell line (Fig. 1g). Among GC cell lines, MKN-45 and AGS showed the highest expression and the lowest expression of circCCDC9, respectively. Thus, we selected MKN-45 and AGS cell lines to investigate the downstream regulatory pathway of circCCDC9.

### Characterization and clinical features of circCCDC9

**Fig. 2:**
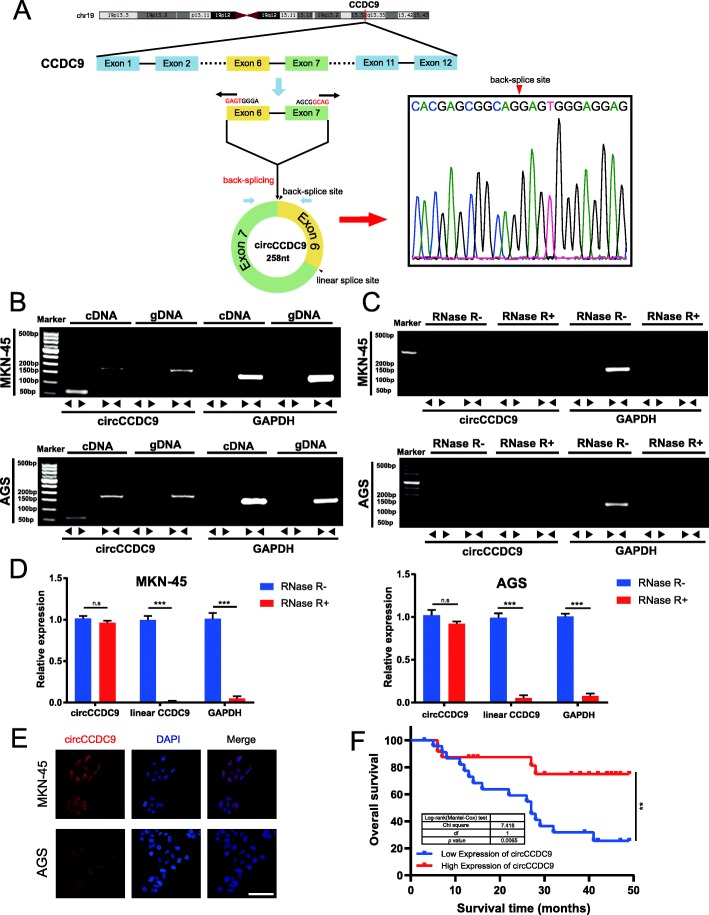
Characterization and clinical features of circCCDC9. **a**. We confirmed the head-to-tail splicing of circCCDC9 in the circCCDC9 RT-PCR product by Sanger sequencing and also determined its genomic size and sequence as reported in the circBase database. **b**. RT-PCR validated the existence of circCCDC9 in MKN45 and AGS cell lines. CircCCDC9 was amplified by divergent primers in cDNA but not gDNA. GAPDH was used as a negative control. **c** and **d**. The expression of circCCDC9 and CCDC9 mRNA in both MKN45 and AGS cell lines was detected by PCR assay followed by nucleic acid electrophoresis or qRT-PCR in the presence or absence of RNase R. **e**. FISH assay showed that circCCDC9 was predominantly localized in the cytoplasm. Nuclei were stained with DAPI for blue color, and cytoplasmic circCCDC9 were stained for red color. (magnification, 400×, scale bar, 100 μm) **f**. Kaplan-Meier survival curves of GC patients with low and high circCCDC9 expression. Using median circCCDC9 value as a cutoff. Data were showed as mean ± SD; ns indicated no significance. **P < 0.01, ***P < 0.001

CircCCDC9 originated from the CCDC9 gene, which located at chromosome 19, consisted of the head-to-tail splicing of exon 6 and exon 7 (47,256,474-47,271,953) (Fig. 2a). However, head-to-tail splicing could be the result of not only trans-splicing but also genomic rearrangements. To rule out these two possibilities, convergent primers for CCDC9 mRNA and special divergent primers to amplify circCCDC9 were designed. cDNA and gDNA were extracted separately from MKN45 and AGS cells and were subjected to PCR and agarose gel electrophoresis assays. The results indicated that circCCDC9 was detected only in cDNA, with no products being detected in the extracted gDNA (Fig. 2b). Subsequently, we confirmed the head-to-tail splicing of circCCDC9 by Sanger sequencing and also determined its genomic size and sequence as reported in the CircBase database (Fig. 2a). Stability was considered as one of the most crucial characteristics of circRNAs [34]. RNase R was employed in the experiments to confirm the stability of circCCDC9. First, PCR and agarose gel electrophoresis assay were performed with specially designed divergent and convergent primers, in which it was found that circCCDC9, rather than linear CCDC9 or GAPDH, resisted digestion by RNase R (Fig. 2c). Moreover, the expression level of back-spliced or canonical forms of CCDC9 with or without RNase R supplementation was detected in the cDNA and gDNA of GC cells by qRT-PCR (Fig. 2d). CircCCDC9 in cDNA was tested with divergent primers, even under RNase R treatment. The opposite result was observed in gDNA PCR products. Additionally, the linear form of CCDC9 could not be amplified by convergent primers, demonstrating that circCCDC9 was not attributable to genomic rearrangements and PCR artefacts. The results of the FISH assay also showed that circCCDC9 was predominately localized in the cytoplasm (Fig. 2e), which indicated circCCDC9 might function in cytoplasm.

Furthermore, when we collected the clinical data of the aforementioned patients, it was found that the expression level of circCCDC9 significantly correlated with tumor size, lymph node invasion and TNM stage of GC tissues (Table 1). Additionally, overall survival (OS) curves were completed by using the Kaplan-Meier method and survival information for the patients registered previously was gathered. Patients who had low levels of circCCDC9 within their GC tissues had significantly shorter overall survival rate (*P* = 0.0065, log-rank, Fig. 2f). To summarize, circCCDC9 was confirmed to be a circular RNA and a stable and significant prognosis marker that was worthy of further exploration.

**Table 1:** Correlation between expression of circCCDC9 and miR-6792-3p and clinicopathological features in 48 GC fresh-frozen tissues

		circCCDC9 expression		*p* value	miR-6792-3p expression		*p* value
Characteristics	Case	low	high		low	high	
All cases	48	39	9		11	37	
Age at surgery(years)				0.697			0.808
< 60	16	14	2		4	12	
≥60	32	25	7		7	25	
Gender				0.433			0.225
Male	32	27	5		9	23	
Female	16	12	4		2	14	
Tumor size (cm)				**0.001**			**0.002**
≥5	28	27	1		2	26	
< 5	20	12	8		9	11	
T grade				0.767			0.088
T1 + T2	9	7	2		4	5	
T3 + T4	39	32	7		7	32	
Lymph node invasion				**0.010**			**0.000**
Negative(N0)	11	6	5		10	1	
Positive(N1-N3)	37	33	4		1	36	
Tumor site				0.885			0.486
Cardiac	17	14	3		5	12	
Non-cardiac	31	25	6		6	25	
TNM stage				**0.006**			**0.011**
I-II	18	11	7		8	10	
III-IV	30	28	2		3	27	
Histological grade				0.775			0.425
Low	30	24	6		8	22	
Middle-High	18	15	3		3	15	

### CircCCDC9 suppresses GC cells proliferation, migration and invasion in vitro

**Fig. 3:**
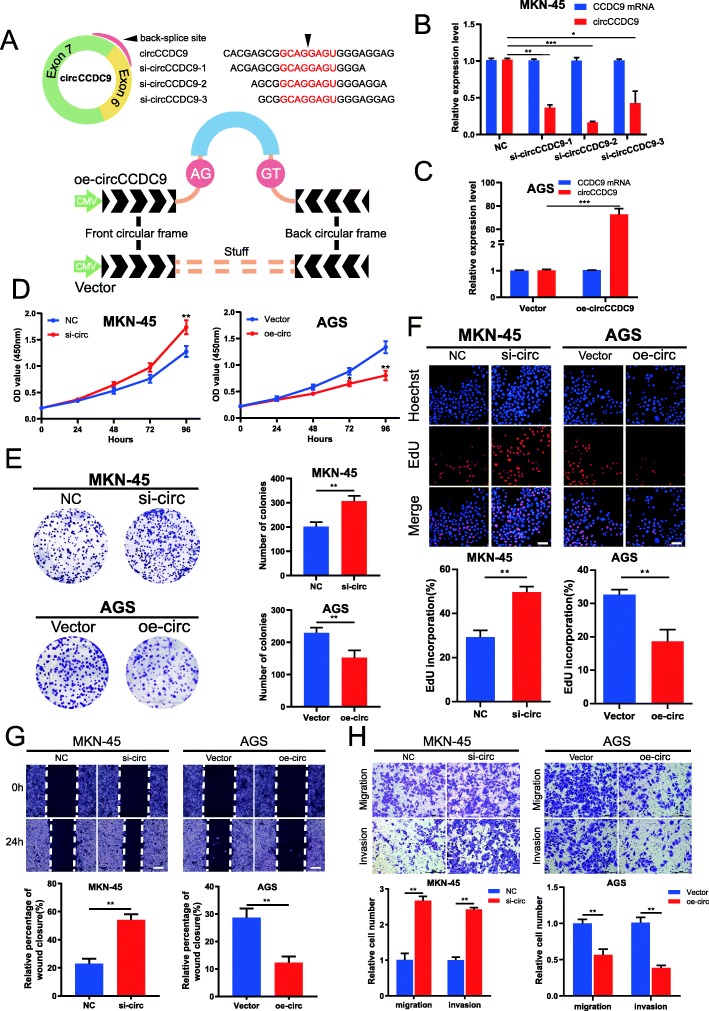
circCCDC9 suppresses GC cells proliferation, migration and invasion in vitro. **a**. The schematic illustration of small interfering RNAs (siRNAs) and circCCDC9 expression vector specifically targeting the backsplice junction sequences. **b**. qRT-PCR analysis of circCCDC9 and CCDC9 mRNA in MKN-45 cells treated with siRNAs. **c**. qRT-PCR analysis of circCCDC9 and CCDC9 mRNA in AGS cells stably overexpressing circCCDC9. **d-f**. CCK-8 assays, colony formation assays and EdU assays were performed to determine the ability of proliferation in MKN45 cells transfected with si-circ or NC and AGS cells transfected with oe-circ or vector. Scale bar, 100 μm. **g** and **h**. Cell migratory and invasive capabilities were assessed by wound healing assays and transwell assays in MKN45 cells transfected with si-circ or NC and AGS cells transfected with oe-circ or vector. The white scale bar indicated 20 μm; the black scale bar indicated 200 μm. Data were showed as mean ± SD. *P < 0.05, **P < 0.01, ***P < 0.001

To explore the biological function of circCCDC9 in GC cells, three siRNA against circCCDC9 and the overexpression vector of circCCDC9 were constructed (Fig. 3a). Three siRNAs were designed to silence circCCDC9 without influencing CCDC9 mRNA level in MKN45 cells. Finally, si-circCCDC9–2 was chosen for the following experiment due to its high inhibitory efficiency (Fig. 3b). The circular transcript expression vector circCCDC9 was successfully constructed in AGS cells (Fig. 3a), as it increased circCCDC9 expression level rather than CCDC9 mRNA (Fig. 3c).

CCK-8 assays demonstrated that the downregulation of circCCDC9 significantly enhanced the proliferation viability, whereas the upregulation of circCCDC9 exerted opposite effects (Fig. 3d). Colony formation assays further demonstrated that the cell cloning capabilities of MKN45 were obviously enhanced by the downregulation of circCCDC9 and markedly impaired by the upregulation of circCCDC9 (Fig. 3e). Similarly, EdU assays revealed that knockdown of circCCDC9 greatly increased the percentages of EdU-positive cells, which considerably decreased at overexpression of circCCDC9 (Fig. 3f). These experiments suggested that circCCDC9 suppressed the proliferation of GC cells.

Then, wound healing and transwell assays were carried out to examine the effects of circCCDC9 on migration and invasion of GC cells. The results indicated that the migratory and invasive capabilities of MKN45 were remarkably enhanced by downregulation of circCCDC9 but significantly suppressed by upregulation of circCCDC9 (Fig. 3g and h). These experiments suggested that circCCDC9 suppressed migration and invasion of GC cells.

### MiR-6792-3p is highly expressed in gastric cancer tissues and correlated with the progression and poor prognosis

According to the theory of competing endogenous RNA (ceRNA), circRNAs function as miRNA sponges and subsequently regulate miRNA expression [16, 18]. Given that circCCDC9 predominantly localized in the cytoplasm and exhibited marked stability, we further explored whether circCCDC9 suppressed the biological behavior of GC by sponging miRNAs. Then, we predicted the potential targets of circCCDC9 by miRNA target prediction tools including circNET, RNAhybrid and miRanda [35–37].

**Fig. 4:**
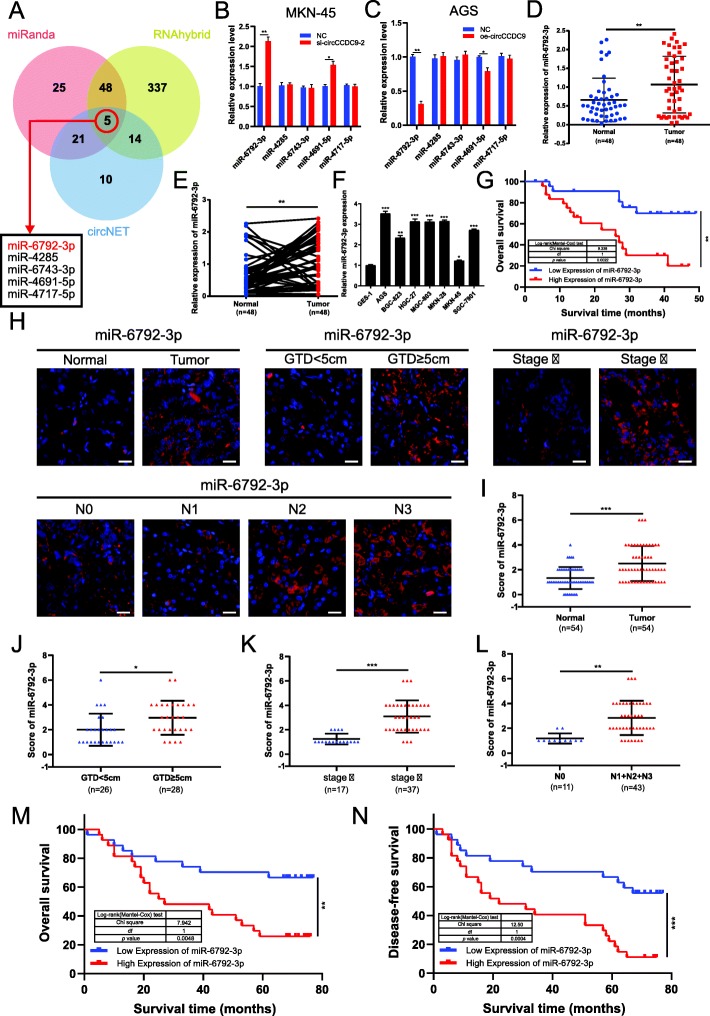
MiR-6792-3p is highly expressed in gastric cancer tissues and correlated with the progression and poor prognosis. **a**. Schematic illustration exhibiting overlapping of the target miRNAs of circCCDC9 predicted by circNET, RNAhybrid and miRanda. **b** and **c**. Relative expression of candidate miRNAs in MKN-45 cells (**b**) and AGS cells (**c**). **d** and **e**. Relative expression of miR-6792-3p in GC tissues and matched adjacent normal tissues was detected by qRT-PCR (*n* = 48). **f**. Relative expression of miR-6792-3p in GC cell lines and normal cell line was detected by qRT-PCR. **g**. Kaplan-Meier plots of the overall survival of GC patients with high and low levels of miR-6792-3p. The cut-off point came from the median value. **h**. Represent images of miR-6792-3p expression in GC tissues with GTD < 5 cm, GTD ≥5 cm, stage II, stage III, N0, N1, N2, N3 and paired adjacent normal tissues. **i**. The level of miR-6792-3p in GC tissues was significantly higher than in paired adjacent normal tissues. **j-l**. The level of miR-6792-3p in GC tissues with GTD ≥5 cm (J), stage III (K) N1, N2 and N3 (L), was significantly higher than those with GTD < 5 cm, stage II and N0, respectively. **m** and **n.** Kaplan-Meier survival analysis (log-rank test) showed that gastric cancer patients with high miR-6792-3p expression had lower OS and DFS than those with low miR-6792-3p expression. The white scale bar indicated 20 μm. Data were showed as mean ± SD. *P < 0.05, **P < 0.01, ***P < 0.001

Overlapping the results of three prediction tools, we selected 5 candidate miRNAs for further validation (Fig. 4a). We compared the levels of candidate miRNAs in MKN-45 cells transfected with si-circCCDC9–2 and NC and AGS cells transfected with circCCDC9 overexpression construct and control vector. Results showed miR-6792-3p, as well as miR-4691-5p, was significantly enhanced in si-circCCDC9–2 group and markedly impaired in circCCDC9 overexpression construct group, compared with other candidates (Fig. 4b and c). Then, we detected the expression of miR-6792-3p and miR-4691-5p both in GC tissues and matched adjacent normal tissues. Results of qRT-PCR showed significant upregulation of miR-6792-3p in GC tissues relative to adjacent normal tissues (Fig. 4d and e), while it showed no significant change of miR-4691-5p level (Fig. S2A and B). We also measured the absolute expression of miR-6792-3p in GC cell lines and tissues by absolution quantification (Fig. S3A-C). Additionally, we detected the relative expression levels of circCCDC9 and miR-6792-3p in MKN-45 cells by the ΔΔCT method (Fig. S3D). These results suggested that the amount of miR-6792-3p was sufficient to mediate the function of circCCDC9. Results of qRT-PCR in GC cell lines also showed significant upregulation of miR-6792-3p relative to GES-1 cells (Fig. 4f). Thus, we selected miR-6792-3p for further study. Then, the relationship between miR-6792-3p expression and clinical characteristics of the GC patients were listed in Table 1. The expression of miR-6792-3p was significantly correlated with tumor size (*P* = 0.002), lymph node invasion (*P* < 0.001) and TNM stage (*P* = 0.011). Kaplan-Meier survival curve indicated that the expression level of miR-6792-3p was reversely correlated with the overall survival of patients with GC (Fig. 4g), indicating miR-6792-3p might be a prognostic marker.

Furthermore, we used another group of gastric cancer tissues in TMA to detect the expression of miR-6792-3p with FISH. Consistent with qRT-PCR results in above 48 paired fresh frozen gastric cancer tissues, the results indicated that the level of miR-6792-3p was significantly higher in gastric cancer tissues than paired adjacent normal tissues (Fig. 4h and i). Further analysis showed that miR-6792-3p was markedly higher in gastric cancer tissues of GTD ≥5 cm (J), stage III (K), N1, N2 and N3 (L) than those with GTD < 5 cm, stage II and N0, respectively (Fig. 4j-l). Moreover, miR-6792-3p expression in gastric cancer tissues was significantly correlated with tumor size, lymph node invasion and TNM stage (Table. S4), which was consistent with previous analysis in fresh-frozen GC tissues. Kaplan-Meier survival curve also showed an inverse correlation between the expression level of miR-6792-3p and the prognosis of GC patients; specifically, GC patients with high miR-6792-3p expression had shorter OS and DFS (Fig. 4m and n). In general, miR-6792-3p acted as a tumor-promoting factor and high miR-6792-3p indicated poor prognosis in GC.

### MiR-6792-3p promotes GC cells proliferation, migration and invasion in vitro by targeting CAV1

**Fig. 5:**
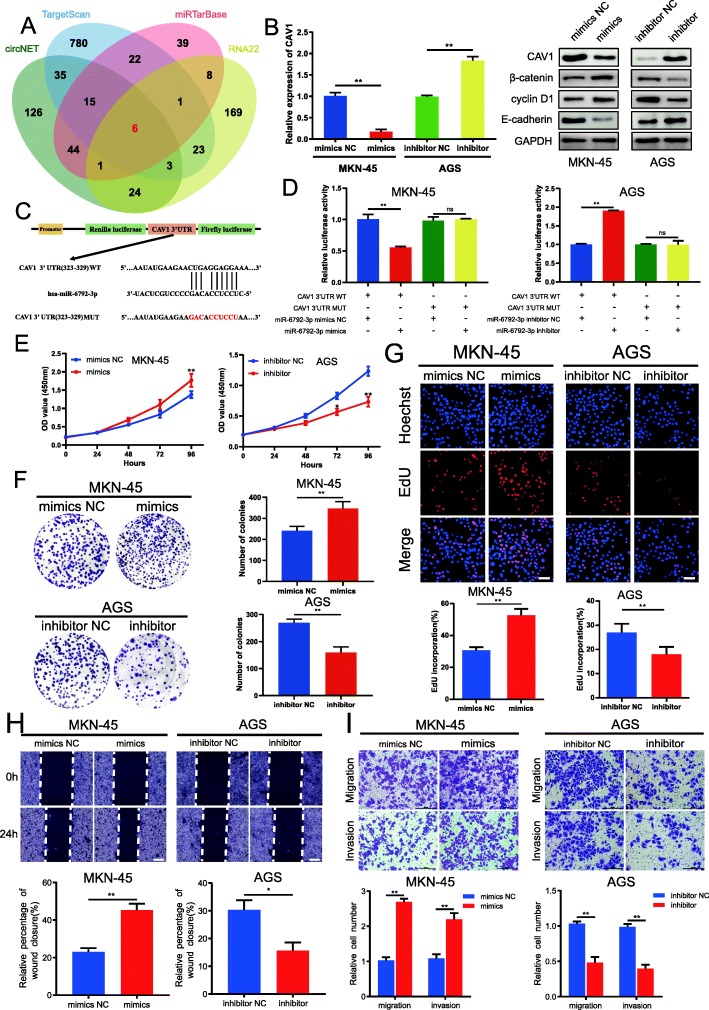
MiR-6792-3p promotes GC cells proliferation, migration and invasion in vitro by targeting CAV1. **a**. Schematic illustration exhibiting overlapping of the target genes of miR-6792-3p predicted by circNET, miRTarBase, RNA22 and TargetScan. **b**. Relative mRNA and protein levels of CAV1 were evaluated by qRT-PCR (left) and western blot (right) in cells transfected with the miR-6792-3p mimics or inhibitor, respectively. **c**. Schematic illustration of CAV1-WT and CAV1-MUT luciferase reporter vectors. **d**. The relative luciferase activities were detected in MKN-45 and AGS cells after co-transfection with CAV1-WT or CAV1-MUT and mimics, inhibitor or NC, respectively. **e-g**. CCK-8 assays, colony formation assays and EdU assays were performed to determine the ability of proliferation in MKN45 cells transfected with mimics or mimics NC and AGS cells transfected with inhibitor or inhibitor NC. Scale bar, 100 μm. **h** and **i**. Cell migratory and invasive capabilities were assessed by wound healing assays and transwell assays in MKN45 cells transfected with mimics or mimics NC and AGS cells transfected with inhibitor or inhibitor NC. The white scale bar indicated 20 μm; the black scale bar indicated 200 μm. Data were showed as mean ± SD; ns indicated no significance.*P < 0.05, **P < 0.01, ***P < 0.001

As few studies had explored the role of miR-6792-3p in GC, we began to clarify the mechanism and biological function of miR-6792-3p in GC cells. Then, we predicted the potential target genes of miR-6792-3p according to circNET, miRTarBase, RNA22 and TargetScan predictions [35, 38–40]. Among candidate target genes including CAV1, NSFL1C, PLCXD3, SLX4, TMEM132C and ZNF629, only CAV1 had been reported to involve the progression of gastrointestinal cancer [27–29] (Fig. 5a). Simultaneously, our previous studies had explored the function and mechanism of CAV1 in gastrointestinal cancer [27–29]. Thus, we selected CAV1 as a target gene of miR-6792-3p for further study. Based on the expression of miR-6792-3p in GC cell lines, AGS cell line showed the relatively higher miR-6792-3p expression level, yet MKN-45 cell line had the relatively lower miR-6792-3p expression level (Fig. 4f). Thus, we transfected miR-6792-3p mimics and inhibitor into MKN-45 cells and AGS cells, respectively, to explore the biological function of miR-6792-3p in GC cells. Results of qRT-PCR displayed substantially increased expression of miR-6792-3p and decreased expression of CAV1 in miR-6792-3p mimics group in MKN-45 cells (Fig. 5b, Fig. S2C) and it also indicated noticeably decreased expression of miR-6792-3p and elevated expression of CAV1 in miR-6792-3p inhibitor group in AGS cells (Fig. 5b, Fig. S2D).

Furthermore, CAV1-WT and CAV1-MUT were cloned into luciferase reporter vector GP-miRGLO and then co-transfected with miR-6792-3p mimics, miR-6792-3p inhibitor or NC into MKN-45 or AGS cells to determine the interaction between miR-6792-3p and CAV1 (Fig. 5c). Results showed that the activity of luciferase reporter vector carrying the CAV1 3’UTR-WT sequence significantly decreased in miR-6792-3p mimics group, but increased in miR-6792-3p inhibitor group. (Fig. 5d). However, these effects disappeared in mutated binding sites of CAV1 (Fig. 5d).

We further explored the biological function of miR-6792-3p in GC cells. Growth curves performed by CCK-8 assays demonstrated that increased expression of miR-6792-3p significantly enhanced the proliferation viability, whereas decreased expression of miR-6792-3p had exerted opposite effects (Fig. 5e). Colony formation assays further demonstrated that the cell cloning capability of MKN45 was obviously improved by increased expression of miR-6792-3p and notably attenuated by decreased expression of miR-6792-3p (Fig. 5f). Similarly, EdU assays revealed that increased expression of miR-6792-3p had markedly increased the percentage of EdU-positive cells, which would obviously be dropped at the decreased expression of miR-6792-3p (Fig. 5g). These experiments suggested that miR-6792-3p promoted the proliferation of GC cells.

Then, wound healing and transwell assays were carried out to examine the effects of miR-6792-3p on migration and invasion of GC cells. The results indicated that the migratory and invasive capabilities of MKN45 were remarkably enhanced by increased expression of miR-6792-3p but significantly impaired by decreased expression of miR-6792-3p (Fig. 5h and i). These experiments suggested that miR-6792-3p promoted migration and invasion of GC cells.

### CircCCDC9 serves as a miRNA sponge of miR-6792-3p to regulate CAV1 expression

**Fig. 6:**
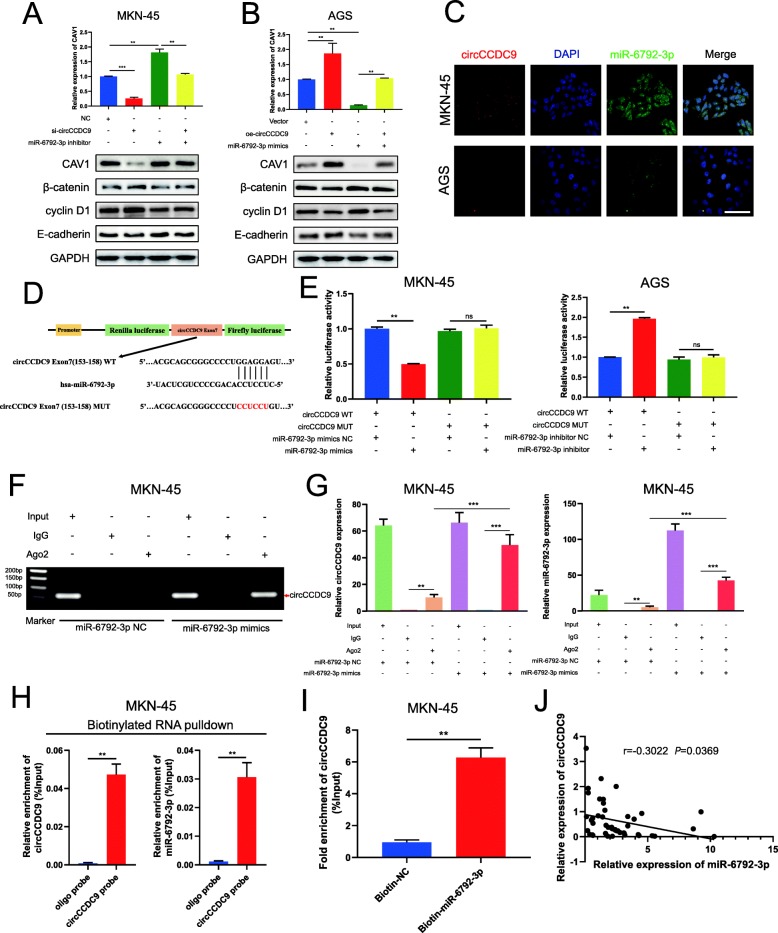
circCCDC9 serves as a miRNA sponge of miR-6792-3p to regulate CAV1 expression. **a** and **b.** Relative mRNA and protein levels of CAV1 were detected in MKN-45 cells transfected with NC, si-circ and inhibitor and AGS cells transfected with NC, oe-circ and mimics using qRT-PCR and western blot, respectively. **c**. FISH was performed to observe the cellular location of circCCDC9 (red) and miR-6792-3p (green) in cells (magnification, 400×, scale bar, 100 μm). **d**. Schematic illustration of circCCDC9-WT and circCCDC9-MUT luciferase reporter vectors. **e**. The relative luciferase activities were detected in 293 T cells after co-transfection with circCCDC9-WT or circCCDC9-MUT and mimics, inhibitor or NC, respectively. **f** and **g.** Anti-Ago2 RIP assay was executed in MKN-45 cells after transfection with mimics and NC, followed by nucleic acid electrophoresis and qRT-PCR to detect circCCDC9 and miR-6792-3p, respectively. **h** and **i**. RNA pull-down assay was executed in MKN-45 cells, followed by qRT-PCR to detect the enrichment of circCCDC9 and miR-6792-3p. **j**. Pearson correlation analysis of the correlation of circCCDC9 with miR-6792-3p expression based on GC tissues. Data were showed as mean ± SD; ns indicated no significance. **P < 0.01, ***P < 0.001

Many researchers reported that circRNA had assumed the role of a miRNA sponge in tumor development regulation [11, 16, 18]. In addition, circRNA could further regulate downstream gene expression. In order to further explore the interaction among circCCDC9, miR-6792-3p and CAV1, we performed a series of experiments. First, qRT-PCR and western blot assays demonstrated that the knockdown of circCCDC9 significantly decreased the mRNA and protein level of CAV1, whereas inhibition of miR-6792-3p increased the above. Also, the co-transfection of si-circCCDC9 and miR-6792-3p inhibitor might eliminate effects in MKN-45 cells (Fig. 6a). Similarly, it was also found that overexpression of circCCDC9 significantly increased the mRNA and protein level of CAV1 and mimics of miR-6792-3p did the opposite effect, whereas co-transfection of oe-circCCDC9 and miR-6792-3p mimics might counteract these effects in AGS cells (Fig. 6b). Second, considering that circRNAs could serve as miRNA sponges in the cytoplasm, FISH assay was performed in MKN-45 and AGS cells to observe the subcellular localization of circCCDC9 and miR-6792-3p. In our research, most of circCCDC9 (red) and miR-6792-3p (green) were co-located in the cytoplasm (Fig. 6c). Third, to confirm the bioinformatics prediction analysis, dual-luciferase reporter assay was applied in GC cells. The full-length of circCCDC9-WT and mutant version without miR-6792-3p binding sites were subcloned into GP-miRGLO plasmids (Fig. 6d). The results indicated the luciferase activity of circCCDC9-WT group significantly decreased in miR-6792-3p mimic group, while increased in miR-6792-3p inhibitor group compared with NC group (Fig. 6e). And there was no difference among miR-6792-3p mimics, inhibitor group and NC group in the luciferase activity of circCCDC9-MUT group. These results suggested that a direct interaction might exist between circCCDC9 and miR-6792-3p. It was widely acknowledged that miRNAs regulated target gene expression by binding to Argonaute 2 (Ago2), an essential component of RNA–induced silencing complex (RISC). Subsequently, RNA immunoprecipitation (RIP) assays were performed in MKN-45 cells to pull down the RNA transcripts that bound to Ago2. Results of agarose gel electrophoresis and qRT-PCR assays indicated that circCCDC9 and miR-6792-3p were all efficiently pulled down by anti-Ago2 as compared with those in the input control. Moreover, circCCDC9 and miR-6792-3p were highly enriched in cells transfected with miR-6792-3p mimics compared with that in NC group (Fig. 6f and g). To further confirm the direct interaction between circCCDC9 and miR-6792-3p, we applied a RNA pull-down assay with biotin-labeled circCCDC9 probe and miR-6792-3p probe. Interestingly, results of qRT-PCR showed that circCCDC9 and miR-6792-3p were highly enriched in circCCDC9 probe group compared with control probe group. In addition, biotin-labeled miR-6792-3p probe captured more circCCDC9 compared with control probe (Fig. 6h and i). Furthermore, results of qRT-PCR in circCCDC9, miR-6792-3p and CAV1 from 48 fresh-frozen GC tissues showed highly positive correlation between circCCDC9 and CAV1, and markedly negative correlation between circCCDC9 and miR-6792-3p, as well as miR-6792-3p and CAV1 (Fig. 6j and Fig. S2E and F). Collectively, these data demonstrated that circCCDC9 acted as a sponge for miR-6792-3p, thus promoting CAV1 expression in GC.

### CircCCDC9 suppresses GC cell proliferation, migration and invasion through circCCDC9/miR-6792-3p/CAV1 axis

**Fig. 7:**
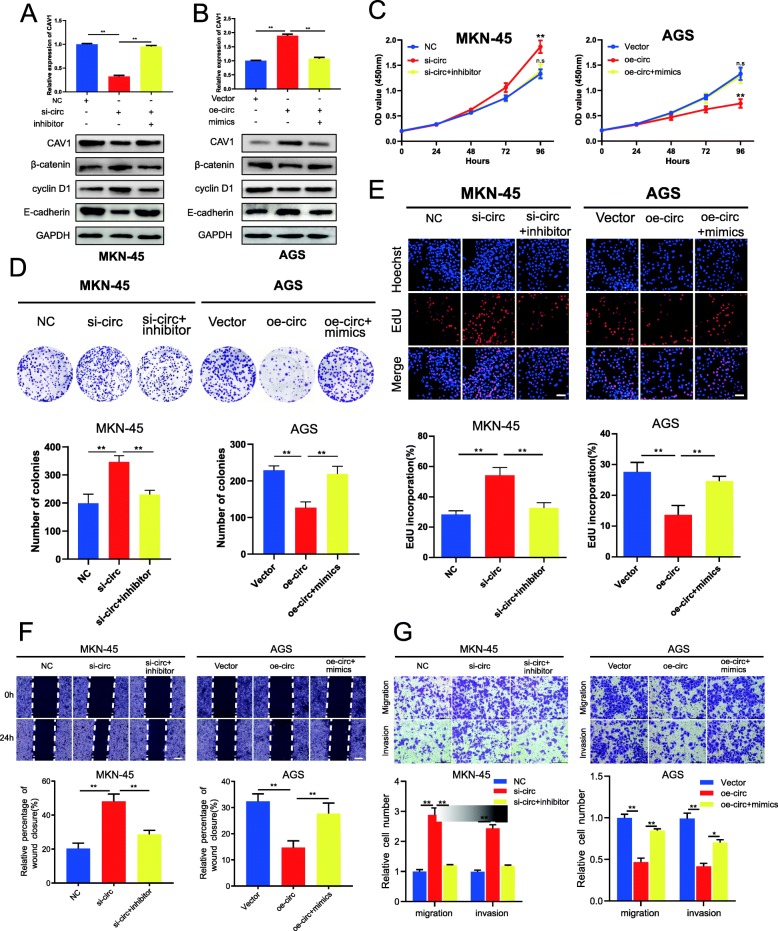
circCCDC9 suppresses GC cell proliferation, migration and invasion through circCCDC9/miR-6792-3p/CAV1 axis. **a** and **b**. Relative mRNA and protein levels of CAV1 in MKN-45 or AGS cells transfected with indicated mimics, inhibitor, NC, si-circ or oe-circ by qRT-PCR and western blot assays, respectively. **c-e**. CCK-8 assays, colony formation assays and EdU assays were performed to determine the ability of proliferation in MKN-45 or AGS cells transfected with indicated mimics, inhibitor, NC, si-circ or oe-circ, respectively. Scale bar, 100 μm. **f** and **g**. Cell migratory and invasive capabilities were assessed by wound healing assays and transwell assays in MKN-45 or AGS cells transfected with indicated mimics, inhibitor, NC, si-circ or oe-circ, respectively. The white scale bar indicated 20 μm; the black scale bar indicated 200 μm. Data were showed as mean ± SD. *P < 0.05, **P < 0.01

To verify whether circCCDC9 served its tumor suppressor function through circCCDC9/miR-6792-3p/CAV1 axis, rescue experiments were designed using miR-6792-3p inhibitor and mimics. Results of qRT-PCR and western blot assays demonstrated that knockdown of circCCDC9 decreased the mRNA and protein levels of CAV1 in MKN-45(Fig. 7a), while upregulation of circCCDC9 enhanced the levels of CAV1 in AGS (Fig. 7b). Simultaneously, the effects caused by silencing or overexpressing circCCDC9 were reversed by miR-6792-3p inhibitor or mimics, respectively (Fig. 7a and b). Moreover, we attempted to explore whether the biological function of circCCDC9 in GC cells could also be reversed by miR-6792-3p inhibitor or mimics. The results indicated that the miR-6792-3p inhibitor reversed the proliferation, migration and invasion promoting effects induced by knockdown of circCCDC9 in MKN-45 cells, whereas miR-6792-3p mimics counteracted the suppressing effects induced by overexpression of circCCDC9 in AGS cells by CCK-8, colony formation, wound healing and Transwell assays (Fig. 7c-g). Collectively, these data demonstrated that circCCDC9 served as a ceRNA for miR-6792-3p to regulate CAV1 expression, thus leading to the progression and development of GC.

### Overexpression of circCCDC9 inhibits the growth and liver metastasis of gastric cancer in vivo

**Fig. 8:**
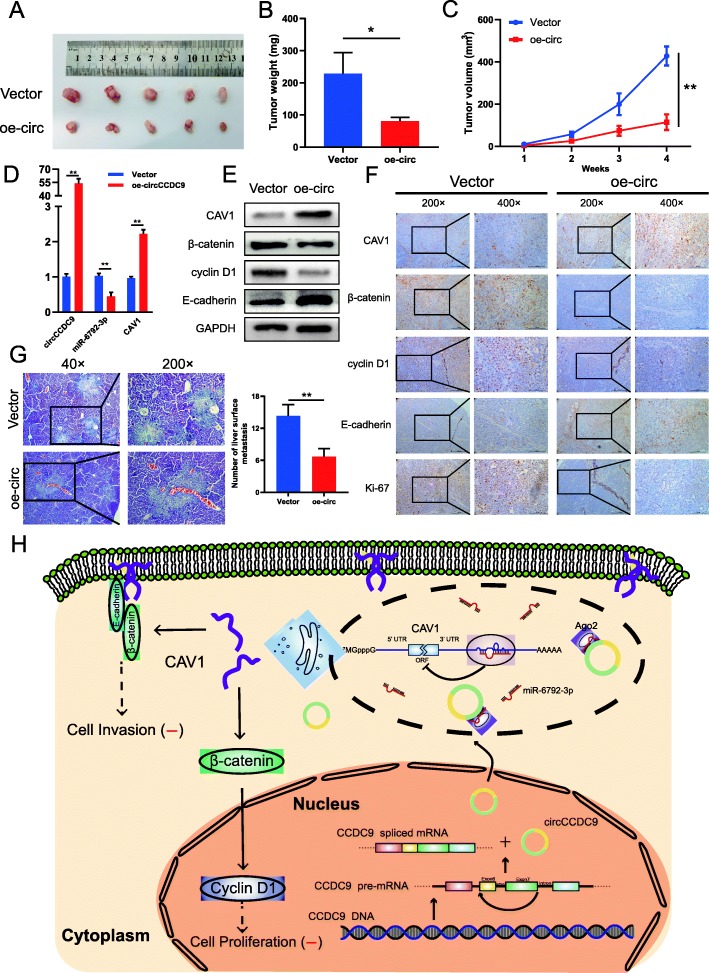
Overexpression of circCCDC9 inhibits the growth of gastric cancer in vivo. **a**. Image of subcutaneous tumor tissues in circCCDC9-overexpressing group and control group. **b**. The relative weights of tumors were evaluated. **c**. Analysis of tumor volume of mice measured every week. **d**. Relative expression levels of circCCDC9, miR-6792-3p and CAV1 were observed in subcutaneous tumor tissues by qRT-PCR. **e**. Relative expression levels of CAV1, β-catenin, cyclin D1 and E-cadherin were observed in subcutaneous tumor tissues by western blot assays. **f**. Relative expression levels of CAV1, β-catenin, cyclin D1 and E-cadherin were observed in subcutaneous tumor tissues by IHC. **g**. Representative images in HE staining of the liver metastasis of AGS cells. **h**. The schematic diagram shows the mechanism underlying circCCDC9 as a ceRNA for miR-6792-3p to regulate CAV1 expression in the progression of gastric cancer. Data were showed as mean ± SD. *P < 0.05, **P < 0.01

To further explore the effects of circCCDC9 on tumor growth and liver metastasis in vivo, AGS cells stably transfected with oe-circCCDC9 or control vector were subcutaneously injected into BALB/c nude mice. Every 7 days, the tumor volumes were measured. After 28 days, the tumor weights were determined. Compared with the control group, the circCCDC9 over-expression group significantly reduced the tumor volume and weight (Fig. 8a-c). These subcutaneous tumor tissues were further deployed for qRT-PCR, WB and IHC assays. And results of qRT-PCR and WB showed the expression of CAV1 was upregulated in circCCDC9 over-expression group (Fig. 8d and e). IHC assays also demonstrated that upregulation of circCCDC9 enhanced the expression of CAV1, E-cadherin and weakened the expression of β-catenin, cyclin D1 and Ki-67 in xenograft tumor tissues (Fig. 8f). To investigate the role of circCCDC9 in tumor metastasis, AGS cells stably transfected with circCCDC9 or control vector were intravenously injected into the ileocolic vein of nude mice. Compared with the control group, the circCCDC9 over-expression group had less liver metastatic nodules (Fig. 8g). Taken together, these findings demonstrated with first lines of evidence that circCCDC9 acted as a miR-6792-3p sponge to suppress the progression of gastric cancer through enhancing CAV1 expression (Fig. 8h).

## Discussion

CircRNAs are a type of non-coding RNAs that have been discovered more than four decades ago [41]. CircRNAs are pervasive but heterogeneous among multiple tissues and cell lines and the generation of circRNAs is regulated by specific *cis*-regulatory elements and *trans*-acting factors [42, 43]. Previous studies considered circRNAs as transcriptional noise and aberrant splicing by-products [5, 6]. However, accumulating evidence about the biogenesis and functions of circRNAs has given rise to a new perspective on understanding the pathogenesis of diverse diseases, including cancer, cardiovascular disease, autoimmune disease and so on [44–46]. Some circRNAs were reported to function as oncogenes or tumor suppressors in diverse human cancers, such as colon cancer, lung cancer, hepatocellular carcinoma (HCC) and gastric cancer [47]. So far, only a few GC-related circRNAs have been well recognized [48]. In the present study, it is the first research shows that circCCDC9/miR-6792-3p/CAV1 pathway participates in GC progression.

In this study, we first revealed a novel circular RNA through circRNA microarray analysis termed circCCDC9 that was obviously downregulated in GC and significantly correlated with tumor size and lymph node invasion as well as overall survival of GC patients. Second, from functional study in vitro and in vivo, we identified that circCCDC9 acted as a tumor suppressor to inhibit the proliferation, metastasis and invasion of GC cells. Third, miR-6792-3p, predicted by bioinformatics analyses and validated by dual luciferase reporter assays, was significantly upregulated in GC cells and tissues and was correlated with patients’ OS and proliferation and metastasis of GC cells. Fourth, mechanistic experiments demonstrated that circCCDC9 functioned as a ceRNA through harboring miR-6792-3p to counteract its suppressive effect on the target gene CAV1 in GC progression. Given the above evidence, our results suggested the importance of circCCDC9 as a novel biomarker and therapeutic target in human GC.

Up to now, circRNAs have been mainly reported on its active role in cytoplasm, where they acted as miRNA sponges, interacted with RBP and involved in protein translation [8–10]. Given that most circRNAs contained miRNA response elements (MREs), accumulating researches shown that the ceRNA mechanism represented the primary approach through which circRNAs perform its biological functions [8, 11, 16, 18, 49]. For example, overexpression of circPRMT5 in urothelial carcinoma of the bladder activated the SNAIL1-induced EMT pathway by blocking the expression of miR-30c, which resulted in tumor metastasis [49]. Moreover, it was reported that circAGFG1, which was significantly upregulated in triple-negative breast cancer, promotes tumor growth and metastasis by upregulating the downstream oncogene CCNE1. Meanwhile, miR-195-5p could silence the expression of CCNE1 gene and inhibit proliferation and metastasis of tumors, whereas circAGFG1 could facilitate this process by inhibiting miR-96-5p [50]. Herein, analyzed by circNET, RNAhybrid and miRanda, circCCDC9 was predicted to harbor miRNA-binding sites of miR-6792-3p. Thus, a series of experiments such as FISH, biotin-labeled probe pull-down assay, dual-luciferase reporter assay and RIP assay, confirmed that circCCDC9 and miR-6792-3p were co-located in the cytoplasm of GC cells and circCCDC9 directly bound to miR-6792-3p. Subsequent rescue experiments further confirmed that circCCDC9 reversed the oncogenic roles of miR-6792-3p. Furthermore, we discovered that upregulated expression of miR-6792-3p suppressed the activation of CAV1 in GC cells, suggesting that miR-6792-3p was a critical negative regulator of the CAV1. Therefore, our results provided sufficient evidence to suggest that circCCDC9 acted as a miR-6792-3p sponge and provided an exploitable biomarker and therapeutic target for patients with GC.

CAV1 has been proved to contribute to multiple malignancies. Our previous studies showed that the tumor suppressor CAV1 regulated the epithelial-mesenchymal transition (EMT) and played critical roles in GC progression [29]. To date, whether and how circRNAs contributed to CAV1-induced progression in GC remains elusive. In the present study, our results indicated that circCCDC9 interacted with miR-6792-3p to promote the expression of CAV1, and inferred a novel mechanistic role of circCCDC9/miR-6792-3p/CAV1 axis in regulating the progression of GC. Strikingly, a recent study indicated CAV1 acted as a stage-specific growth modulator, which served as a suppressor in the early stage but shifted its function in the later stage of GC [51]. However, our study indicated CAV1 served as a tumor suppressor in GC. It was speculated that the reason for this might be different tumor microenvironments in different stages or cell-types [52]. Therefore, further studies are necessary to obtain a better understanding of the function of CAV1 in tumor progression.

A recent study also revealed CAV1 depletion could stabilize HER2 at the cell surface, further to increase the tumor avidity for trastuzumab on HER2-positive gastric cancer [53, 54]. Thus, we explored the role of circCCDC9/miR-6792-3p/CAV1 axis on HER2 expression in GC. Our findings in this study had supported previous research that the negative correlation between CAV1 expression and HER2 expression (Fig. S4). In addition, our results also shown the negative correlation between circCCDC9 expression and HER2 expression, suggested that circCCDC9 might affect HER2 expression by CAV1, at least partially. Hence, further studies are necessary to explore the underlying mechanism between circCCDC9 and HER2 in GC.

To our knowledge, this is the first study that thoroughly investigates the expression, regulation, function and clinical implication of circCCDC9 in GC. Moreover, this is also the first research to study the relationship between miR-6792-3p and CAV1. These findings may bring light to the treatment of GC. However, there are several limitations to the interpretation of our study results. Firstly, GC tissues used for RNA-seq in our study were taken from a homogenous population from one hospital, we could not rule out the possibility that there might be other critical downregulated circRNAs which are also involved in CAV1-induced progression in GC. Secondly, our preliminary study suggested that circCCDC9 was significantly downregulated in GC tissues. So, further investigation of circCCDC9 in body fluids is also needed, which makes it possible for circCCDC9 to be an ideal biomarker and therapeutic target in GC. Thirdly, our study demonstrated the ability of circCCDC9 to bind to miR-6792-3p. Whereas, there might be other miRNAs that were bound to circCCDC9 to regulate the progression and development of GC, which were not predicted by bioinformatics analyses. Fourth, whether circCCDC9 regulated the development of GC through other mechanisms such as interacting with RNA-binding proteins and sponging trans-acting elements required further investigation. Therefore, a deeper understanding of the therapeutic potential of circCCDC9 in GC is in need of further exploration.

## Conclusion

In summary, our findings provided the first line of comprehensive evidence that circCCDC9 was obviously downregulated in GC and functioned as a tumor suppressor, as well as a prognostic biomarker in GC. Furthermore, we also demonstrated that circCCDC9 exerted a bona fide regulatory role in blocking the activity of miR-6792-3p, thereby participating in the CAV1-induced pathways to inhibit GC cell proliferative and aggressive capacities, as shown in Fig. 8h. Clearly, our results not only explained the potential mechanisms related to circRNA in the regulation of GC cell progression but also provided a novel potential therapeutic target for patients with GC.

## Supplementary information

**Additional file 1: Table S1.** Primers used in this study. **Table S2.** Oligonucleotides and probes used in this study.

**Additional file 2: Figure S1.** Relative expression of circCCDC9 in gastric cancer tissues by ISH using TMA of 54 pairs of gastric cancer and adjacent normal tissues. **A.** Represent images of circCCDC9 expression in GC tissues with GTD < 5 cm, GTD ≥5 cm, stage II, stage III, N0, N1, N2, N3 and paired adjacent normal tissues. **B.** The level of circCCDC9 in GC tissues was significantly lower than that in paired adjacent normal tissues. **C-E.** The level of circCCDC9 in GC tissues with GTD ≥5 cm (**C**), stage III(**D**), N1, N2, N3 (**E**) was significantly lower than those with GTD < 5 cm, stage II and N0, respectively. **F&G**. Kaplan-Meier survival analysis (log-rank test) showed that gastric cancer patients with high circCCDC9 expression had longer OS and DFS than those with low circCCDC9 expression. The white scale bar indicated 50 μm. Data were showed as mean ± SD. *P < 0.05, **P < 0.01, ***P < 0.001.

**Additional file 3: Table S3.** Correlation between circCCDC9 expression and clinicopathological features in 54 GC tissues from TMA

**Additional file 4: Figure S2.** Relative expression of miR-6792-3p and miR-4691-5p. **A&B.** Relative expression of miR-4691-5p in GC tissues and matched adjacent normal tissues was detected by qRT-PCR (*n* = 48). **C&D.** Relative expression of miR-6792-3p in cells transfected with the miR-6792-3p mimics or inhibitor, respectively. **E&F.** Pearson correlation analysis of the correlation of circCCDC9 with CAV1 expression and miR-6792-3p with CAV1 based on GC tissues, respectively. Data were showed as mean ± SD. **P < 0.01, ***P < 0.001.

**Additional file 5: Table S4** Correlation between miR-6792-3p expression and clinicopathological features in 54 GC tissues from TMA.

**Additional file 6: Figure S3.** The absolute expression of miR-6792-3p in GC tissues and cell lines. **A.** Standard curve of miR-6792-3p in absolute quantification. **B.** Copy number of miR-6792-3p in 20 pg RNA of GC cell lines. **C.** Copy number of miR-6792-3p in 20 pg RNA of GC tissues and adjacent normal tissues. **D.** The relative expression of circCCDC9 and miR-6792-3p in MKN-45 cell line. Data were showed as mean ± SD. **P<0.01, ***P<0.001.

**Additional file 7: Figure S4.** Correlation between circCCDC9 and HER2 in GC. **A.** Relative expression of HER2 in GC tissues was detected by qRT-PCR (n = 48). **B&C.** Pearson correlation analysis of the correlation of CAV1 with HER2 expression and circCCDC9 with HER2 expression based on GC tissues, respectively. **D.** Relative protein levels of CAV1 and HER2 in AGS cells transfected with Vector, oe-circ. **E.** IF was performed to observe the expression and location of HER2 in AGS cells transfected with Vector, oe-circ.

## Data Availability

The datasets used in current study are available from the corresponding author on reasonable request.

## Abbreviations

CircRNA: Circular RNA
qRT-PCR: Real-time quantitative polymerase chain reaction
RIP: RNA immunoprecipitation
ISH: In situ hybridization
miRNA: MicroRNA
MREs: MicroRNA response elements
CAV1: Caveolin 1
TMA: Tissue microarray
AJCC: American Joint Commission on Cancer
UICC: The Union for International Cancer Control
GTD: Greatest tumor diameter
PVDF: Polyvinylidene fluoride
IHC: Immunohistochemical analysis
OS: Overall survival
ceRNA: competing endogenous RNA
3’UTR: 3′-untranslated region
