# Supplementary material for: Circular RNA circCCDC9 acts as a miR-6792-3p sponge to suppress the progression of gastric cancer through regulating CAV1 expression
Source: Mol Cancer. 2020 May 9;19:86. doi: 10.1186/s12943-020-01203-8 (PMC7210689; doi:10.1186/s12943-020-01203-8)
Supplement: Supplementary file 1 — Additional file 1: Table S1. Primers used in this study. Table S2. Oligonucleotides and probes used in this study. [file 12943_2020_1203_MOESM1_ESM.pdf]

Table S1 Primers used in this study

Name	Primer	Sequence (5'-3')
circCCDC9	Forward primer	CTGTGGCCTTGAGCACGAG
	Reverse primer	CATACTCGGCGATCTTCTCCA
linear CCDC9	Forward primer	GAAGACCGATGGGATGTTCAAG
	Reverse primer	GACAGGAACTCCCCAAAAGTAG
GAPDH	Forward primer	GGGAAGGTGAAGGTCGGAGT
	Reverse primer	GGGGTCATTGATGGCAACA
miR-6792-3p	Stem-loop Primer	GTCGTATCCAGTGCAGGGTCCGAGG TATTCGCACTGGATACGACATGAGC
	Forward primer	CGCTCCTCCACAGCCCCT
	Reverse primer	AGTGCAGGGTCCGAGGTATT
miR-6743-3p	Stem-loop Primer	GTCGTATCCAGTGCAGGGTCCGAGG TATTCGCACTGGATACGACTGTGGG
	Forward primer	CGAGCCGCTCTTCTCCCTG
	Reverse primer	ATCCAGTGCAGGGTCCGAGG
miR-4691-5p	Stem-loop Primer	GTCGTATCCAGTGCAGGGTCCGAGG TATTCGCACTGGATACGACCCGCAG
	Forward primer	CGGTCCTCCAGGCCATGAG
	Reverse primer	ATCCAGTGCAGGGTCCGAGG
miR-4717-5p	Stem-loop Primer	GTCGTATCCAGTGCAGGGTCCGAGG TATTCGCACTGGATACGACACACAT
	Forward primer	CGTAGGCCACAGCCACCC
	Reverse primer	AGTGCAGGGTCCGAGGTATT
miR-4285	Stem-loop Primer	GTCGTATCCAGTGCAGGGTCCGAGG TATTCGCACTGGATACGACATGAGT
	Forward primer	CGGCGGCGAGTCCG
	Reverse primer	ATCCAGTGCAGGGTCCGAGG
U6	Stem-loop Primer	CTCAACTGGTGTCGTGGAGTCG GCAATTCAGTTGAGAAAAATAT
	Forward primer	CAAGGATGACACGCAAA
	Reverse primer	TCAACTGGTGTCGTGG
CAV1	Forward primer	AATACTGGTTTTACCGCTTGCT
	Reverse primer	CATGGTACAACTGCCCAGATG

Table S2 Oligonucleotides and probes used in this study

Definition	Sequence (5'-3')
si-circCCDC9-1	Sense: ACGAGCGGCAGGAGUGGGATT<br>Antisense: UCCCACUCCUGCCGCUCGUGC
si-circCCDC9-2	Sense: AGCGGCAGGAGUGGGAGGATT<br>Antisense: UCCUCCCACUCCUGCCGCUCG
si-circCCDC9-3	Sense: GCGGCAGGAGUGGGAGGAGTT<br>Antisense: CUCCUCCCACUCCUGCCGCUC
si-circCCDC9 NC	Sense: UUCUCCGAACGUGUCACGUTT<br>Antisense: ACGUGACACGUUCGGAGAATT
miR-6792-3p mimics	Sense: CUCCUCCACAGCCCCUGCUCAU<br>Antisense: GAGCAGGGGCUGUGGAGGAGUU
miR-6792-3p mimics NC	Sense: UUCUCCGAACGUGUCACGUTT<br>Antisense: ACGUGACACGUUCGGAGAATT
miR-6792-3p inhibitor	AUGAGCAGGGGCUGUGGAGGAG
miR-6792-3p inhibitor NC	CAGUACUUUUGUGUAGUACAA
Biotin-circCCDC9 probe	TCCTCCCACTCCTGCCGCTCG
Biotin-miR-6792-3p	ATGAGCAGGGGCTGTGGAGGAG
Oligo probe	GTGTAACACGTCTATACGCCCA
Cy3-labeled circCCDC9 probe	CCGCTCCTCCCACTCCTGCCGCTCGT
FAM-labeled miR-6792-3p probe	ATGAGCAGGGGCTGTGGAGGAG
Digoxin-labeled circCCDC9 probe	CCGCTCCTCCCACTCCTGCCGCTCGT
