# Supplementary figures and images for: Circular RNA circCCDC9 acts as a miR-6792-3p sponge to suppress the progression of gastric cancer through regulating CAV1 expression
Source: Mol Cancer. 2020 May 9;19:86. doi: 10.1186/s12943-020-01203-8 (PMC7210689; doi:10.1186/s12943-020-01203-8)

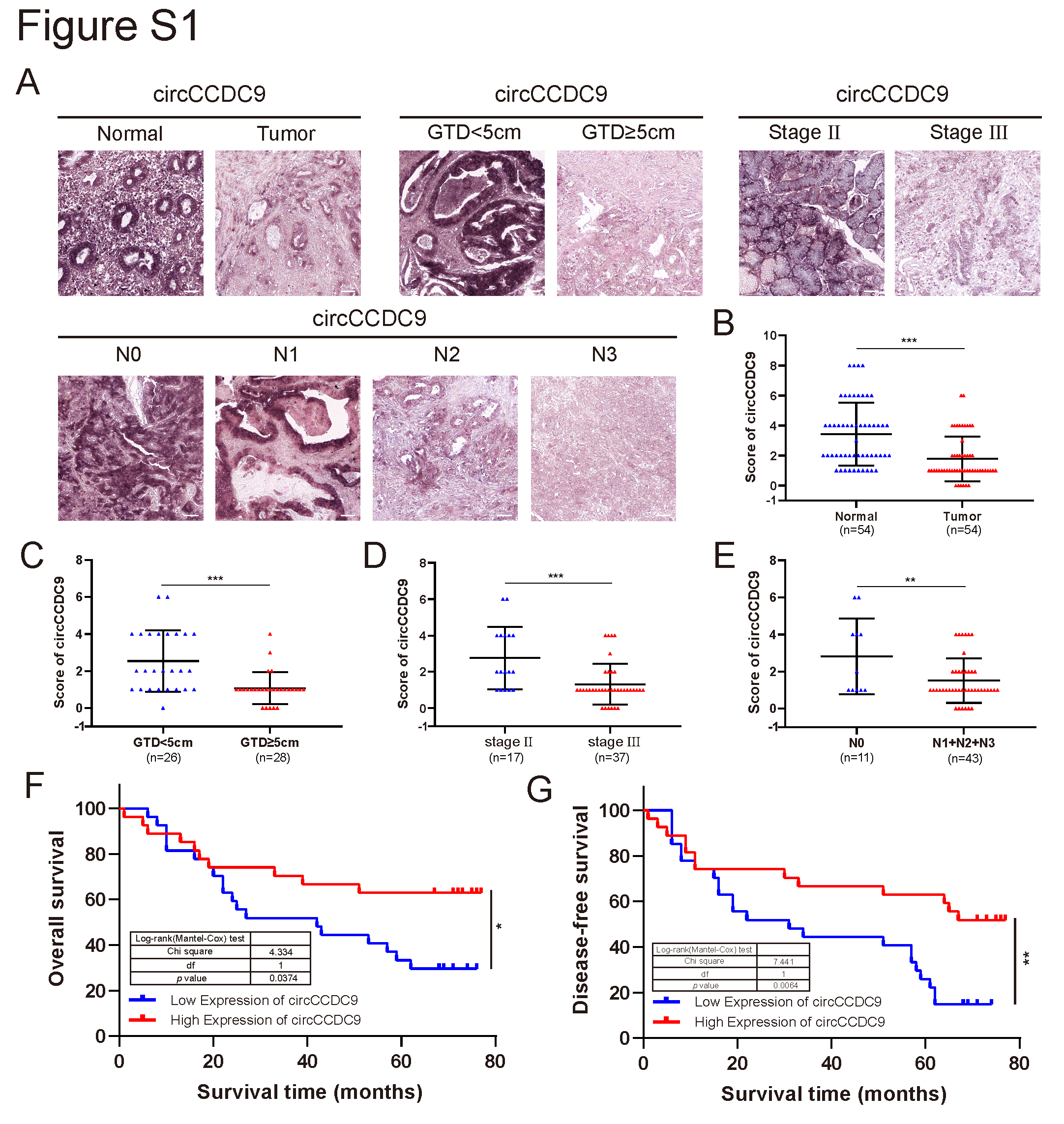

Supplement: Supplementary file 2 — Additional file 2: Figure S1. Relative expression of circCCDC9 in gastric cancer tissues by ISH using TMA of 54 pairs of gastric cancer and adjacent normal tissues. A. Represent images of circCCDC9 expression in GC tissues with GTD < 5 cm, GTD ≥5 cm, stage II, stage III, N0, N1, N2, N3 and paired adjacent normal tissues. B. The level of circCCDC9 in GC tissues was significantly lower than that in paired adjacent normal tissues. C-E. The level of circCCDC9 in GC tissues with GTD ≥5 cm (C), stage III(D), N1, N2, N3 (E) was significantly lower than those with GTD < 5 cm, stage II and N0, respectively. F&G. Kaplan-Meier survival analysis (log-rank test) showed that gastric cancer patients with high circCCDC9 expression had longer OS and DFS than those with low circCCDC9 expression. The white scale bar indicated 50 μm. Data were showed as mean ± SD. *P < 0.05, **P < 0.01, ***P < 0.001. [file 12943_2020_1203_MOESM2_ESM.tiff]

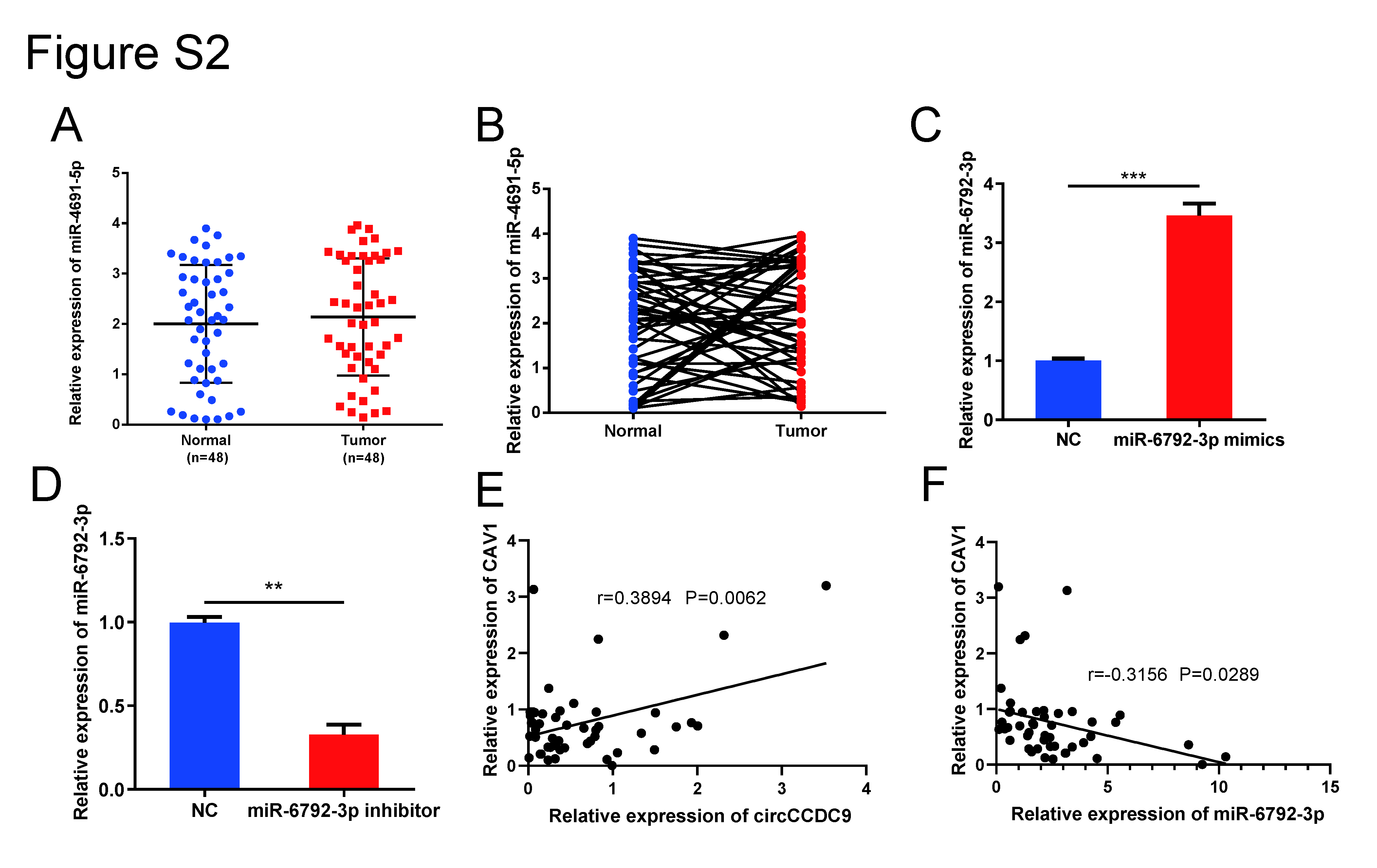

Supplement: Supplementary file 4 — Additional file 4: Figure S2. Relative expression of miR-6792-3p and miR-4691-5p. A&B. Relative expression of miR-4691-5p in GC tissues and matched adjacent normal tissues was detected by qRT-PCR (n = 48). C&D. Relative expression of miR-6792-3p in cells transfected with the miR-6792-3p mimics or inhibitor, respectively. E&F. Pearson correlation analysis of the correlation of circCCDC9 with CAV1 expression and miR-6792-3p with CAV1 based on GC tissues, respectively. Data were showed as mean ± SD. **P < 0.01, ***P < 0.001. [file 12943_2020_1203_MOESM4_ESM.tiff]

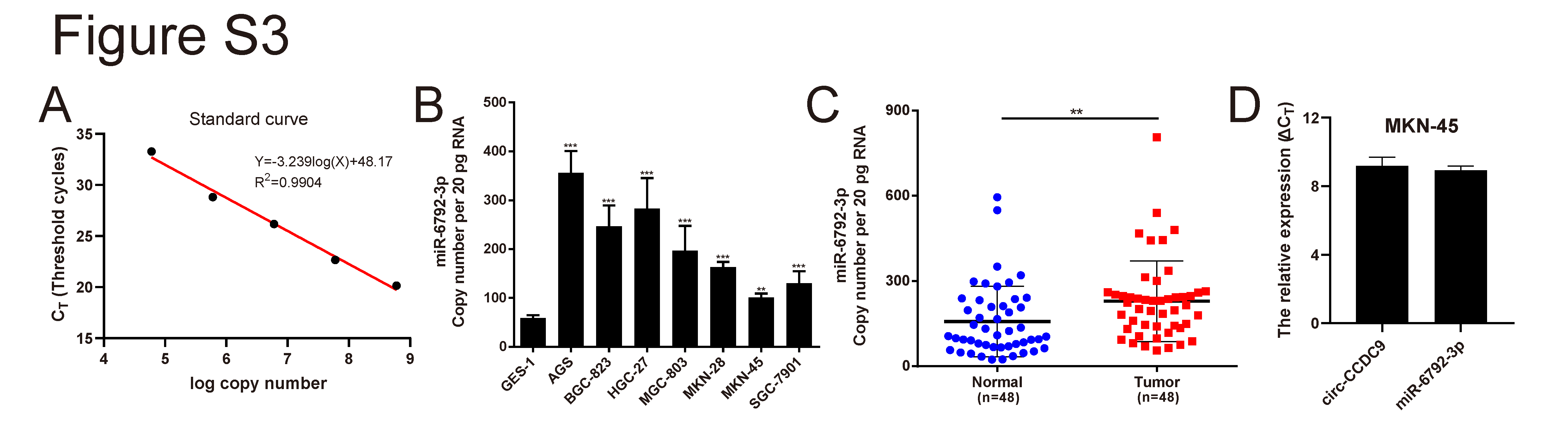

Supplement: Supplementary file 6 — Additional file 6: Figure S3. The absolute expression of miR-6792-3p in GC tissues and cell lines. A. Standard curve of miR-6792-3p in absolute quantification. B. Copy number of miR-6792-3p in 20 pg RNA of GC cell lines. C. Copy number of miR-6792-3p in 20 pg RNA of GC tissues and adjacent normal tissues. D. The relative expression of circCCDC9 and miR-6792-3p in MKN-45 cell line. Data were showed as mean ± SD. **P<0.01, ***P<0.001. [file 12943_2020_1203_MOESM6_ESM.tiff]

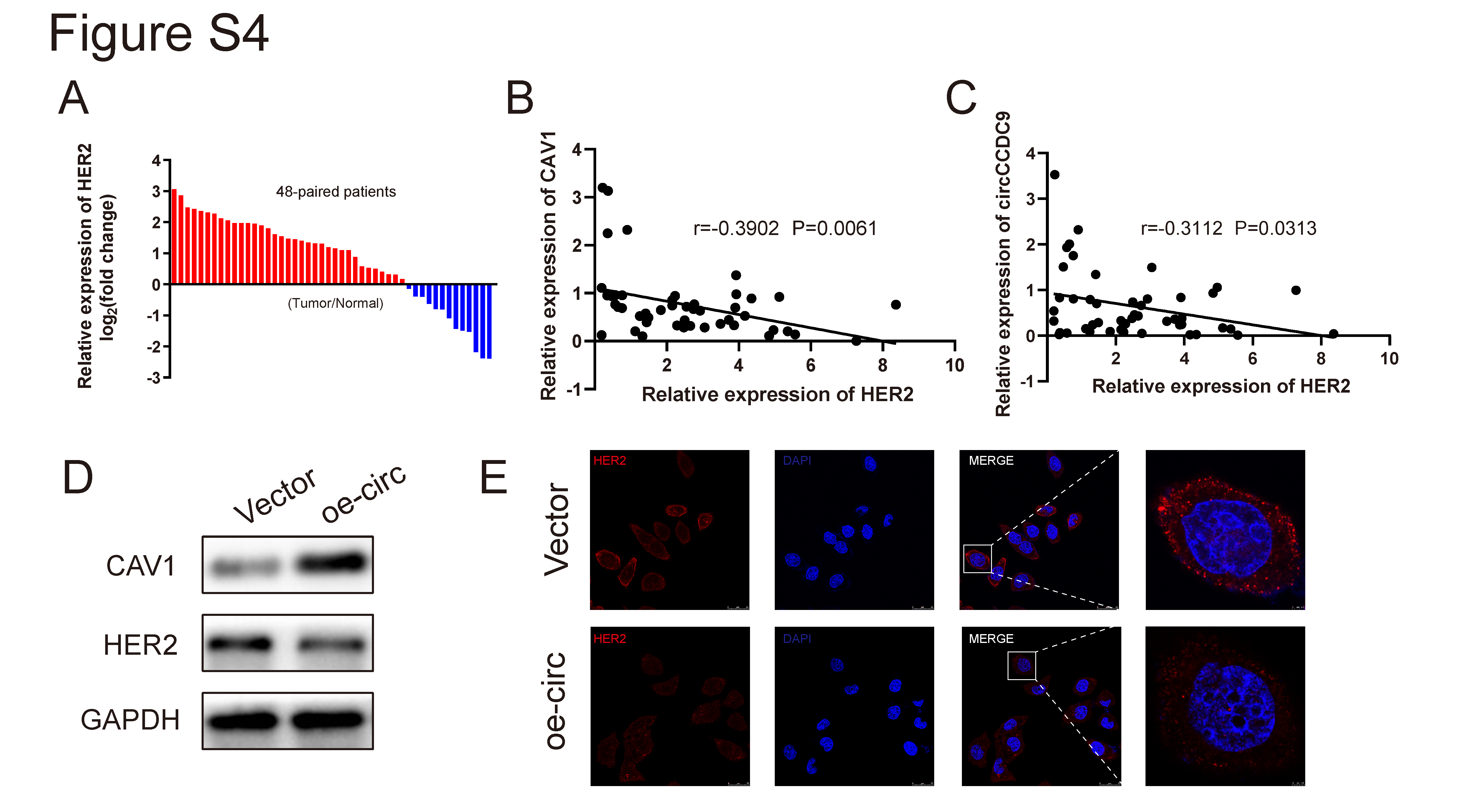

Supplement: Supplementary file 7 — Additional file 7: Figure S4. Correlation between circCCDC9 and HER2 in GC. A. Relative expression of HER2 in GC tissues was detected by qRT-PCR (n = 48). B&C. Pearson correlation analysis of the correlation of CAV1 with HER2 expression and circCCDC9 with HER2 expression based on GC tissues, respectively. D. Relative protein levels of CAV1 and HER2 in AGS cells transfected with Vector, oe-circ. E. IF was performed to observe the expression and location of HER2 in AGS cells transfected with Vector, oe-circ. [file 12943_2020_1203_MOESM7_ESM.tiff]
