# Supplementary material for: Circular RNA circCCDC9 acts as a miR-6792-3p sponge to suppress the progression of gastric cancer through regulating CAV1 expression
Source: Mol Cancer. 2020 May 9;19:86. doi: 10.1186/s12943-020-01203-8 (PMC7210689; doi:10.1186/s12943-020-01203-8)
Supplement: Supplementary file 3 — Additional file 3: Table S3. Correlation between circCCDC9 expression and clinicopathological features in 54 GC tissues from TMA [file 12943_2020_1203_MOESM3_ESM.pdf]

Table S3 Correlation between circCCDC9 expresssion and clinicopathological features in 54 GC tissues from TMA.

Characteristics	Case	CircCCDC9 expression		*p* value
		low	high	
Tumor cases	54	40	14	
Age at surgery(years)				1.000
<60	15	11	4	
≥60	39	29	10	
Gender				0.208
Male	38	30	8	
Female	16	10	6	
GTD (cm)				**0.043**
≥5	28	24	4	
<5	26	16	10	
T stage				0.595
T2	5	3	2	
T3+T4	49	37	12	
Lymph node invasion				**0.000**
Negative(N0)	11	1	10	
Positive(N1-N3)	43	39	4	
Tumor site				1.000
Cardiac	13	10	3	
Non-cardiac	41	30	11	
TNM stage				**0.000**
II	17	5	12	
III	37	35	2	
Histological grade				0.657
Low	32	23	9	
Middle-High	22	17	5	

GTD：Greatest tumor diameter.
