# Supplementary material for: Circular RNA circCCDC9 acts as a miR-6792-3p sponge to suppress the progression of gastric cancer through regulating CAV1 expression
Source: Mol Cancer. 2020 May 9;19:86. doi: 10.1186/s12943-020-01203-8 (PMC7210689; doi:10.1186/s12943-020-01203-8)
Supplement: Supplementary file 5 — Additional file 5: Table S4 Correlation between miR-6792-3p expression and clinicopathological features in 54 GC tissues from TMA. [file 12943_2020_1203_MOESM5_ESM.pdf]

Table S4 Correlation between miR-6792-3p expresssion and clinicopathological features in 54 GC tissues from TMA.

Characteristics	Case	miR-6792-3p expression		*p* value
		low	high	
Tumor cases	54	16	38	
Age at surgery(years)				0.333
<60	15	6	9	
≥60	39	10	29	
Gender				0.411
Male	38	10	28	
Female	16	6	10	
GTD (cm)				**0.010**
≥5	28	4	24	
<5	26	12	14	
T stage				0.627
T2	5	2	3	
T3+T4	49	14	35	
Lymph node invasion				**0.000**
Negative(N0)	11	11	0	
Positive(N1-N3)	43	5	38	
Tumor site				0.170
Cardiac	13	6	7	
Non-cardiac	41	10	31	
TNM stage				**0.000**
II	17	14	3	
III	37	2	35	
Histological grade				0.770
Low	32	9	23	
Middle-High	22	7	15	

GTD：Greatest tumor diameter.
